# Investigation of Safety Profile of Four* Copaifera* Species and of Kaurenoic Acid by* Salmonella*/Microsome Test

**DOI:** 10.1155/2019/7631531

**Published:** 2019-01-10

**Authors:** Jaqueline Lopes Damasceno, Yadira Fernández Arnet, Giovanna Capaldi Fortunato, Luiza Girotto, Gabriel Davi Marena, Beatriz Patti Rocha, Flávia Aparecida Resende, Sergio Ricardo Ambrosio, Rodrigo Cássio Sola Veneziani, Jairo Kenupp Bastos, Carlos Henrique Gomes Martins

**Affiliations:** ^1^Laboratory of Research in Applied Microbiology (LAPEMA), University of Franca, Avenida Dr. Armando Salles de Oliveira 201, 14404-600 Franca, SP, Brazil; ^2^Department of Biological Sciences and Health, University of Araraquara, Rua Carlos Gomes 1338, 14801-320 Araraquara, SP, Brazil; ^3^Department of Pharmaceutical Sciences, School of Pharmaceutical Sciences, University of São Paulo, 14040-903 Ribeirão Preto, SP, Brazil

## Abstract

Trees of the* Copaifera* genus are native to the tropical regions of Latin America and Western Africa.* Copaifera* sp is widely used as a popular medicine and it has various ethnopharmacological indications, including gonorrhea, bronchitis, asthma, skin ulcers, ulcers, sore throat, uterine infections, general inflammations, cancer, and leishmanioses. Kaurenoic acid is a naturally occurring diterpene found in* Copaifera* and has been used as an anti-inflammatory, treatment of ulcer, leishmaniasis, and cancer. Bearing in mind the fact that the Ames test is an excellent tool to assess the safety of extracts, oils, and phytochemicals isolated from medicinal plants, from it, we evaluate the mutagenic potential of four species, between oleoresins (*C. oblongifolia*;* C. langsdorffii*) and leaves extracts (*C. lucens*;* C. multijuga*), of the* Copaifera* genus and also of kaurenoic acid, which is one of its major compounds. The results showed that the* Copaifera* spp. and kaurenoic acid did not induce an increase in the number of revertant colonies, without mutagenic effect in experiments, in the all concentrations evaluated by Ames test. The results obtained in our study support the safe use of the* Copaifera* genus medicinal plants selected and of kaurenoic acid.

## 1. Introduction

Along history, different cultures have used plants for medicinal purposes. Indeed, plants have proven to be a source of medicines for the treatment of a broad spectrum of diseases. Today, plant-based systems continue to play an essential role in health [1, 2] and interest in phytomedicinal products has increased worldwide, so much so that plants are still being investigated as a source of novel medicinal agents [3].

Trees belonging to the genus* Copaifera* are native to the tropical regions of Latin America and Western Africa. The genus* Copaifera* belongs to the family Leguminosae and encompasses 72 species. Over 20* Copaifera *spp. exist in the Brazilian territory, where they are called “copaibeiras”, “pau d'óleo”, or “copaíbas” [4].* Copaifera* spp. are widely employed in popular medicine. They have various ethnopharmacological indications, like treatment of gonorrhea, bronchitis, asthma, skin ulcers, ulcers, sore throat, uterine infections, general inflammations, cancer, and leishmaniases [5–7].

The scientific literature contains numerous reports on the pharmacological activities of* Copaifera* species, such as their anti-inflammatory [4], antitumor [8], antiproliferative [9], anthelmintic [10], antitubercular [11], gastroprotective [12], chemopreventive [13], immunomodulatory [14], and antibacterial [9, 15, 16] actions, among others.

Kaurenoic acid [ent-kaur-16-en-19-oic acid] is a diterpene that occurs naturally in some Brazilian plants, including* Copaifera* oleoresins. Countless pharmacological properties have been reported for kaurenoic acid, such as its anti-inflammatory effect, its use to treat ulcer, and its antiparasitic, analgesic, and anticancer potential [17–19].

Because natural compounds have been traditionally used, they are often assumed to be safe. However, many studies have reported that several plant species applied in traditional medicine exhibit mutagenic, carcinogenic, or toxic effects [20–22]. Nevertheless, a number of plants and phytotherapic products continue being applied without scientific evidence of their safety.

The Ames test is globally known for its ability to spot point mutations caused by different agents. This test employs indicative* Salmonella *Typhimurium strains that are sensitive to substances that induce distinct types of mutations. On the basis of the Ames test, it is possible to establish the mutagenic action of a compound as a function of the* S.* Typhimurium concentration [23, 24]. This assay is applied for initial screening of the mutagenic potential of new drugs worldwide. A mutagenic response has high predictive value for carcinogenicity [25, 26]. Over the years, the scientific community and government agencies and corporations have recognized the value of this assay [27–29].

Bearing in mind that the Ames test is an excellent tool to assess the safety of extracts, oils, and phytochemicals isolated from medicinal plants, we used this test to evaluate the mutagenic potential of the oleoresins or leaf extracts of four* Copaifera *species and of kaurenoic acid.

## 2. Materials and Methods

### 2.1. Plant Material

The plant material was collected in different Brazilian states between August 2012 and May 2014. Plant vouchers were identified either by Dr. Regina Celia Vianna Martins da Silva of the botanical laboratory of the Brazilian Agricultural Research Corporation (Embrapa), Belém, State of Pará, Brazil, or by Dr. Milton Groppo Junior of the Biology Department of the University of São Paulo, Ribeirão Preto Campus, State of São Paulo, Brazil, where the vouchers were deposited.  Table 1 lists information about the voucher specimens.

To draw the* C. oblongifolia* and* C. langsdorffii* oleoresins, an auger was used to drill a hole with diameter of approximately one inch. The hole was drilled in the center of the tree trunk, three feet above the ground. The oleoresin was drained into an amber bottle by means of a pipe connected to a filter. After the oleoresin was collected, the hole was properly sealed [14].

*C. lucens *and* C. multijuga* leaves were air-dried at 40°C for 48 h or lyophilized and powdered in a blender. The obtained powder was submitted to maceration in ethanol/water 7:3 at room temperature for 48 h. After filtration, the solvent was evaporated below 40°C under vacuum. This procedure was repeated four times, and the extracts were combined, concentrated under vacuum, and lyophilized, which provided an average of 20% w/w of leaf crude hydroalcoholic extracts [30].

**Figure 1:**
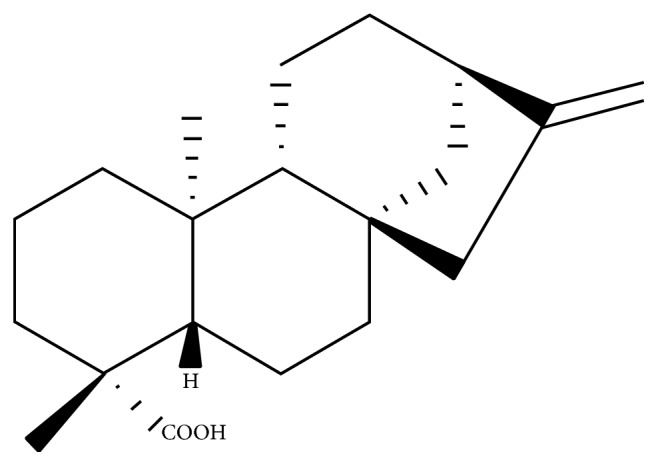
Chemical structure of kaurenoic acid.

Kaurenoic acid (Figure 1), purity above 99%, was isolated as detailed by Simão et al. [31]. The* Copaifera* species oleoresins and leaves were collected and the research was developed after authorization by the Brazilian government through SISBIO (Biodiversity Information and Authorization System #35143-1) and CGEN (Genetic Heritage Management Council #010225/2014-5).

### 2.2. Ames Test

The Ames test was used to investigate* Copaifera* spp. mutagenicity. The preincubation methodology developed by Maron and Ames [23], with and without exogenous activation (S9), was employed to analyze different* Salmonella *Typhimurium strains (TA98, TA100, TA97a, and TA102) in an attempt to identify agents that cause gene mutations. The tester strains, kindly provided by Dr. B.N. Ames (Berkeley, CA, USA), were grown from frozen cultures for 12–14 h, overnight, in Oxoid Nutrient Broth Number 2.

For the mutagenic activity assay, various concentrations of each oleoresin, each extract, or kaurenoic acid dissolved in DMSO were added to 0.1 mL of bacterial culture in 0.5 mL of phosphate buffer 0.2 M or 0.5 mL of 4% S9 mixture and incubated at 37°C for 20–30 min. The concentrations ranged from 62.5 to 500 *μ*g/plate for the* C. lucens *(extract), from 120 to 1000 *μ*g/plate for the* C. multijuga *(extract), from 125 to 1000 *μ*g/plate for the* C. oblongifolia *(oleoresin), 500 to 4000 *μ*g/plate for the* C. langsdorffii* (oleoresin), and from 25 to 200 *μ*g/plate for the kaurenoic acid. These concentrations were selected on the basis of a preliminary toxicity test. In all the subsequent assays, the upper limit of the tested dose range was either the highest nontoxic dose or the lowest toxic dose determined in the preliminary assay. Toxicity was detected either as a reduction in the number of histidine revertants (His+) or as a thinning of the auxotrophic background lawn.

The metabolic activation mixture (S9 fraction) prepared from the livers of* Sprague Dawley* rats treated with the polychlorinated biphenyl mixture Aroclor 1254 (500 mg/kg) was purchased from Molecular Toxicology Inc. (Boone, NC, USA) and freshly prepared before each test. The metabolic activation system consisted of 4% S9 fraction, 1% of magnesium chloride 0.4 M, 1% of potassium chloride 1.65 M, 0.5% of D-glucose-6-phosphate disodium 1 M, and 4% of nicotinamide adenine dinucleotide phosphate sodium salt (NADP) 0.1 M in 50% of phosphate buffer 0.2 M and 39.5% of sterile distilled water.

After incubation, 2 mL of top agar was added, and the mixture was poured onto a plate containing minimal agar. The plates were incubated at 37°C for 48 h, and the His+ revertant colonies were counted manually.

Results were analyzed with the statistical software package Salanal 1.0 (U.S. Environmental Protection Agency, Monitoring Systems Laboratory, Las Vegas, NV, from Research Triangle Institute, RTP, NC, USA); the model of Bernstein et al. [32] was adopted. The data (revertants/plate) were assessed by analysis of variance (ANOVA), followed by linear regression. The mutagenic index (MI) was also calculated for each tested concentration and corresponded to the average number of revertants per test plate divided by the average number of revertants per solvent control plate. A sample was considered mutagenic when a dose-response relationship was detected and MI was higher than two (MI > 2) at one or more concentrations [33, 34].

The following standard mutagens were used as positive controls in experiments without S9 mix: 4-nitro-*O*-phenylenediamine (10 *μ*g/plate) for TA98 and TA97a, sodium azide (1.25 *μ*g/plate) for TA100, and mitomycin C (0.5 *μ*g/plate) for TA102. In experiments with S9 activation, 2-anthramine (1.25 *μ*g/plate) was used as positive control for TA98, TA97a, and TA100, and 2-aminofluorene (10 *μ*g/plate) was employed as positive control for TA102. DMSO served as the solvent control (100 *μ*L/plate) and the negative control corresponds to the rate of spontaneous reversion of each strain.

## 3. Results

Table 2 shows the mean number of revertants/plate (M), the standard deviation (SD), and the mutagenic index (MI) observed for* S.* Typhimurium strains TA98, TA100, TA102, and TA97a in the presence (+S9) or in the absence (-S9) of metabolic activation after sample treatment with the target oleoresin, extract, or compound.

Neither the* C. lucens *and* C. multijuga* leaf extracts nor the* C. langsdorffii* and* C. oblongifolia* oleoresins caused genetic mutations, as evidenced by the Ames test. Kaurenoic acid did not increase the number of revertant colonies, either, so it did not exert mutagenic effects at any of the assayed concentrations or on any of the evaluated strains. The solvent control (DMSO) did not differ significantly of revertants number from the negative control.

## 4. Discussion

The mutagenic effects exerted by plants are not easily noticeable in humans, and adverse long-term outcomes such as cancer can manifest. Thereby, the scientific literature has highlighted the importance of screening medicinal plants for their mutagenic potency [26, 35–37]. In this sense, here we have examined the* Copaifera* spp. and kaurenoic acid mutagenic potential with the aid of the Ames test. Akyıl and Konuk [38] emphasized that genotoxic agent detection often relies on the use of bacteria as test organisms. In this way, the Ames test (or* Salmonella*/microsome test) is the method that is most commonly used to detect genotoxic agent mutagenic effects [23, 38, 39].

The performance of the Ames test using different strains is of great importance considering the peculiarities of each of them in relation to the test. In this way, the* hisG46 *marker in strain TA100 results from the substitution of a leucine (GAG/CTC) by a proline (GGG/CCC). This mutation is reverted to the wild-type state by mutagens that cause base-pair substitution mutations primarily at one of the GC pairs. The* hisD3052 *mutation carried by strain TA98 is a -1 frameshift mutation which affects the reading frame of a nearby repetitive –C–G–C–G–C–G–C–G– sequence. Reversion of the* hisD3052 *mutation back to the wild-type state is induced by various frameshift mutagens such as 2-nitrofluorene and various aromatic nitroso derivatives of amine carcinogens. The* hisD6610 *mutation in strain TA97a also carries a +1 frameshift mutation (cytosine) resulting in a run of 6 cytosines (–C–C–C–C–C–C–). This strain is believed to be more sensitive to some of the mutagens that revert strain TA98. Strain TA102 was developed that contain AT base pairs at the* hisG428 *mutant site. The mutation is carried on the multicopy plasmid pAQ1. The plasmid confers tetracycline resistance, which is a convenient marker to detect the presence of the plasmid. The* hisG428 *mutation is an ochre mutation, TAA, in the* hisG *gene which can be reverted by all six possible base-pair changes; both transitions and transversions. This mutation is also reverted by mutagens that cause oxidative damage, besides to detect cross-linking agents [40].

In addition, a biologically active chemical can be biotransformed into an inactive metabolite. Similarly, an inactive chemical can be biotransformed into an active metabolite [38, 41]. Hence, it is important to use the S9 fraction in the Ames test: it allows analyses to be performed in the presence of metabolism, thereby providing more reliable results.

Herein, regarding safety, in our findings neither kaurenoic acid nor the investigated plants (extracts and oleoresins) exerted mutagenic effects in the different strains of* Salmonella* Typhimurium irrespective of S9 activation.

Most of the papers about the genus* Copaifera* report on oleoresins removed from the tree trunk. However, studying leaf extracts is also relevant because they contain promising bioactive molecules. Indeed, the search for the cure of diseases through leaf infusion may have been one of the first ways of using natural products, a practice that is still adopted nowadays [17].

Many* Copaifera* spp. are popularly employed as medicinal plants in different countries because these species present numerous pharmacological properties. As for kaurenoic acid, several biological effects also have been reported [18, 19, 42].

Our study is the first to investigate on the safety of the* C. lucens* and* C. oblongifolia *species and also to employ* C. langsdorffii* in oleoresin for the study of mutagenicity. The effects of* C. multijuga* (oleoresin/extract) on DNA were addressed in previous studies, however, employing different techniques in relation to our study that used the Ames test. Thus, our results corroborate with data published by other authors, who tested other species of* Copaifera* and their chemical constituents, or used different experimental models, and demonstrated that they do not damage DNA.

In this way, the oleoresin of* C. multijuga* and its chemical marker, diterpene copalic acid, were evaluated by Alves et al. [13] through the micronucleus assay (V79 cell) and the Ames test for* in vitro* study, as well as micronucleus and comet assays (Swiss mice) for* in vivo* assay. The data obtained showed that none of them exert no genotoxic/mutagenic effect under the experimental conditions employed. When compared to our results, these data indicate that for* C. multijuga* both the extract, which was evaluated in our study, and oleoresin, as evaluated by Alves et al. [13], do not affect the number of revertant colonies compared to the negative control in Ames test; the same applies to copalic acid and kaurenoic acid. These findings suggest that mutagenicity is absent, regardless of metabolic activation.

In a recent study Furtado et al. [30] evaluated the genotoxic potential of* C. multijuga* and the results demonstrated absence of damage to DNA, in view of that the treatment both with oleoresin and the leaf extract of* C. multijuga* does not significantly increased micronucleus frequency* in vitro* (V79 cell) and* in vivo* (Swiss mice). In addition, the authors also evaluated extracts and oleoresins from other species of this genus, such as* C. duckei*,* C. reticulata*,* C. paupera *and* C. pubiflora* and as well as the results found for* C. multijuga*, the absence of genotoxicity was reported for all species tested.

The results obtained in studies of Alves et al. [43] and Batista et al. [44] demonstrated that* C. langsdorffii* extract did not significantly increase the frequency of micronuclei (Swiss mice) in peripheral blood and bone marrow, respectively. In other study, the comet assay using Wistar rats did not reveal any significant differences between animals treated with the* C. langsdorffii* extract only and the negative control group [45]. These data showing that the extract does not display genotoxicity.

Recently,* in vivo *micronucleus test and comet assay using Wistar rats showed that the* Copaifera malmei *extract is not genotoxic and has antimutagenic activity. Moreover, the subchronic toxicity test did not reveal toxicologically relevant changes, as judged from behavioral, biochemical, and hematological analyses for up to 30 days. These results pointed to the* Copaifera malmei *extract high safety margin for therapeutic use [46]. Toxicity and genotoxicity determinations evidenced that Copaiba oil use is also safe: histopathological evaluation did not reveal changes in Copaiba oil-treated animals, and mutagenicity assessment (micronucleus test; 2000 mg/kg b.w.) did not show genotoxic effects [47].

Leandro et al. [16] used the Ames test to show that the* C. trapezifolia *extract is not mutagenic against the same* Salmonella *Typhimurium strains tested herein, independent of metabolic activation.

In relation to the various* Copaifera *species chemical composition, UPLC-MS/MS and CG/MS analyses of the oleoresins have identified acid diterpenes and major volatile sesquiterpenes, whereas high contents of phenolic compounds including flavonoid heterosides and galloylquinic acid derivatives have been verified in the leaves [30]. Among the oleoresin constituents, diterpenes are by far the main components and include* ent*-agathic acid,* ent*-copalic acid, and* ent*-kaurenoic acid, followed by sesquiterpenes like *β*-bisabolene, *α*-humulene, and trans-*β*-caryophyllene [30, 48]. In the case of* Copaifera *species leaf hydroalcoholic extracts, they contain mainly quercetin, afzelin, and quinic acids [30].

According to Almeida et al. [49], the Copaiba oleoresin (commercial product) and its fractions, which contain sesquiterpenes, methyl esters of diterpene carboxylic acid, and high *β*-caryophyllene levels, are not genotoxic as evidenced by* in vivo* comet assay or micronucleus test. *β*-caryophyllene, the main constituent of oleoresins and volatile fractions, does not promote cytotoxic or genotoxic effects in human lymphocyte cultures, and it protects against DNA damage induced by ethyl methane sulfonate [50]. Evaluation of nine sesquiterpenes, including trans-caryophyllene, by the Ames test has shown that none of the compounds are mutagenic [51].

In a recent study, treatment of gastric cancer and normal stomach mucosa cell lines with kaurenoic acid showed that the acid concentration strongly correlates with the DNA damage index and with the micronucleus frequency, as determined by comet assay and micronucleus test, respectively [25]. On the other hand, Cavalcanti et al. [36] reported that low concentrations of kaurenoic acid, a bioactive diterpenoid extracted from* C. langsdorffii*, does not exert DNA damage or alter micronucleus frequency in V79 cells, either. Significantly increased DNA damage became evident only after cell exposure to higher kaurenoic acid concentrations (30 or 60 *μ*g/mL).

Here, we determined the kaurenoic acid toxicity to each evaluated* Salmonella *Typhimurium strain by using acid concentrations starting from the toxicity limit. Higher kaurenoic acid concentrations prevent bacterial growth, which enabled us to assess the mutagenic potential of this compound. On the basis of our results, the oleoresins tested herein are not mutagenic even at the highest assayed concentrations.

According to the literature, the use of different organisms or diverse test systems can provide distinct results [16, 35]. This is because genotoxicity and mutagenicity test systems are divided into two groups. Cytogenetic methods analyze eukaryotes and give information that varies from gene mutation to chromosome damage and aneuploidies. In contrast, bacterial methods analyze prokaryotes and afford information about gene mutation and primary DNA damage caused by an agent [52].

Thus, tests like sister-chromatid exchange, chromosomal aberration, and micronucleus have been applied to detect DNA damage at the chromosomal level in human biomonitoring [53–55] whilst the Ames* Salmonella*/microsome mutagenicity assay has been extensively employed to verify the mutagenic activity of countless chemical substances and crude plant extracts [40, 56, 57].

According to Ferguson [58], substances may be clastogenic in the case of mammalian cells, which is the case of substances used in the micronucleus test. However, these same substances may test negative in bacterial assays such as the Ames test. Thus, it is important to evaluate the safety of plants or their chemical compounds focusing the evaluating the different kinds of genetic damage. The association of the Ames test with* in vitro* mammalian cell studies is recommended because they can cover several essential mutagenic parameters (genetic mutations, structural chromosome damage, and aneuploidy) and also cover the tests in prokaryotic and eukaryotic systems. In addition, the literature also highlights that the study by the Ames test should not be omitted because the bacterial gene-mutation test detects all relevant modes of action specifically leading to gene mutations [59].

Previous work observed that compounds may be exclusively positive in one or more of the mammalian cell lines, that is, the positive results were not supporting from the Ames test or* in vivo *tests [60]. In fact, the results obtained first by the Ames test are subsequently reproduced in tests using animals [40]; therefore, absence of mutagenicity in the Ames test has allowed new drugs with fewer side effects to be produced [61, 62]. These data highlight the importance of studies such as ours, demonstrating the absence of plant mutagenicity and its main components, using the Ames test.

## 5. Conclusions

Overall, our results support the safe use of the selected medicinal plants belonging to the genus* Copaifera*. Nevertheless, the mutagenic effects of single compounds could be masked due to antagonistic effects of other compounds present in extracts or oleoresins [26]. Thus, our findings also demonstrate that both kaurenoic acid and the evaluated medicinal plants can be considered potentially safe for therapeutic use.

## Figures and Tables

**Table 1:** Information about the collected *Copaifera* species.

***Copaifera* species**	**Location (City/State)**	**Herbarium**	**Identification number**
***Oleoresins***			
***C. langsdorffii***	Cajuru/SP	SPFR^1^	14438
***C. oblongifolia***	Cajuru/SP	SPFR	14437
***Leaves extract***			
***C. multijuga***	Manacapuru/AM	SPFR	180069
***C. lucens***	Macujaí/PR	EMBRAPA^2^	474303

1 SPFR: Faculty of Philosophy, Sciences and Letters of Ribeirão Preto, Department of Biology, Ribeirão Preto, São Paulo; 2 EMBRAPA: Brazilian Agricultural Research Corporation (Embrapa Eastern Amazon).

**(a):** 

***Copaifera lucens*** ***(extract)***	**Number of revertants (M ± SD)/ plate and MI**							
	**TA98**		**TA100**		**TA97a**		**TA102**	
***μ*** **g/plate**	**-S9**	**+S9**	**-S9**	**+S9**	**-S9**	**+S9**	**-S9**	**+S9**

**C-**	15 ± 3	20 ± 2	140 ± 13	90 ± 15	106 ± 18	135 ± 14	255 ± 33	327 ± 40
**DMSO**	12 ± 1	18 ± 6	133 ± 5	86 ± 10	109 ± 13	143 ± 8	236 ± 22	312 ± 38
**62.5**	17 ± 3 (1.42)	28 ± 1 (1.50)	140 ± 14 (1.06)	115 ± 9 (1.33)	123 ± 12 (1.12)	149 ± 9 (1.04)	272 ± 25 (1.15)	394 ± 33 (1.26)
**125**	14 ± 4 (1.13)	21 ± 3 (1.15)	141 ± 10 (1.06)	124 ± 4 (1.44)	111 ± 14 (1.02)	137 ± 12 (0.96)	215 ± 13 (0.91)	360 ± 21 (1.15)
**250**	16 ± 2 (1.33)	23 ± 5 (1.25)	139 ± 18 (1.04)	129 ± 13 (1.50)	108 ± 8 (0.98)	142 ± 17 (1.00)	240 ± 26 (1.02)	330 ± 29 (1.06)
**375**	13 ± 1 (1.08)	22 ± 6 (1.20)	139 ± 19 (1.04)	126 ± 6 (1.46)	87 ± 4 (0.79)	138 ± 12 (0.97)	246 ± 23 (1.04)	335 ± 23 (1.07)
**500**	10 ± 2 (0.83)	22 ± 2 (1.17)	118 ± 9 (0.89)	124 ± 10 (1.44)	82 ± 11 (0.75)	147 ± 11 (1.03)	262 ± 10 (1.11)	323 ± 37 (1.03)
**C+**	432 ± 23 ^a^	875 ± 45 ^d^	1480 ± 82 ^b^	1151 ± 63 ^d^	1760 ± 95 ^a^	1985 ± 114 ^d^	1520 ± 118 ^c^	2251 ± 156 ^e^

**(b):** 

***Copaifera multijuga*** ***(extract)***	**Number of revertants (M ± SD)/ plate and MI**							
	**TA98**		**TA100**		**TA97a**		**TA102**	
***μ*** **g/plate**	**-S9**	**+S9**	**-S9**	**+S9**	**-S9**	**+S9**	**-S9**	**+S9**

**C-**	15 ± 3	20 ± 2	140 ± 13	90 ± 15	106 ± 18	135 ± 14	255 ± 33	327 ± 40
**DMSO**	12 ± 1	18 ± 6	133 ± 5	86 ± 10	109 ± 13	143 ± 8	236 ± 22	312 ± 38
**120**	14 ± 1 (1.17)	22 ± 5 (1.17)	135 ± 12 (1.02)	117 ± 11 (1.36)	106 ± 2 (0.97)	170 ± 18 (1.19)	262 ± 31 (1.11)	378 ± 20 (1.21)
**250**	15 ± 5 (1.21)	19 ± 2 (1.01)	135 ± 8 (1.01)	115 ± 8 (1.34)	103 ± 8 (0.94)	181 ± 17 (1.27)	255 ± 12 (1.08)	381 ± 24 (1.22)
**500**	16 ± 3 (1.33)	20 ± 1 (106)	134 ± 3 (1.01)	106 ± 6 (1.23)	97 ± 15 (0.89)	155 ± 20 (1.09)	244 ± 26 (1.03)	346 ± 16 (1.11)
**750**	14 ± 2 (1.17)	23 ± 2 (1.25)	111 ± 6 (0.83)	96 ± 8 (1.12)	86 ± 11 (0.79)	169 ± 19 (1.18)	215 ± 22 (0.91)	313 ± 22 (1.00)
**1000**	16 ± 1 (1.33)	16 ± 1 (0.85)	109 ± 5 (0.82)	98 ± 11 (1.14)	76 ± 7 (0.70)	141 ± 18 (0.99)	204 ± 13 (0.86)	310 ± 18 (0.99)
**C+**	432 ± 23 ^a^	875 ± 45 ^d^	1480 ± 82 ^b^	1151 ± 63 ^d^	1760 ± 95 ^a^	1985 ± 114 ^d^	1520 ± 118 ^c^	2251 ± 156 ^e^

**(c):** 

***Copaifera oblongifolia*** ***(oleoresin)***	**Number of revertants (M ± SD)/ plate and MI**											
	**TA98**			**TA100**				**TA102**			**TA97a**	
***μ*** **g/plate**	**- S9**	***μ*** **g/plate**	**+ S9**	***μ*** **g/plate**	**- S9**	***μ*** **g/plate**	**+ S9**	***μ*** **g/plate**	**- S9**	**+ S9**	**- S9**	**+ S9**

**C-**	14 ± 3		20 ± 4		103 ± 15		137 ± 11		310 ± 35	257 ± 29	128 ± 12	134 ± 17
**DMSO**	12 ± 1	**0.0** ^a^	15 ± 1	**0.0** ^a^	118 ± 8	**0.0** ^a^	132 ± 7	**0.0** ^a^	310 ± 12	305 ± 27	123 ± 13	117 ± 25
**125**	15 ± 4 (1.33)	**31.25**	20 ± 3 (1.30)	**125**	124 ± 4 (1.06)	**31.25**	113 ± 8 (0.86)	**12.5**	349 ± 12 (1.13)	350 ± 21 (1.15)	154 ± 19 (1.25)	148 ± 15 (1.26)
**250**	15 ± 4 (1.30)	**62.5**	19 ± 1 (1.23)	**250**	130 ± 14 (1.11)	**62.5**	150 ± 16 (1.14)	**25**	383 ± 24 (1.24)	298 ± 20 (0.98)	141 ± 16 (1.15)	168 ± 25 (1.43)
**500**	13 ± 5 (1.13)	**125**	18 ± 1 (1.17)	**500**	108 ± 11 (0.92)	**125**	122 ± 5 (0.92)	**50**	264 ± 17 (0.85)	277 ± 28 (0.91)	149 ± 23 (1.21)	164 ± 16 (1.40)
**750**	12 ± 1 (1.04)	**187.5**	18 ± 4 (1.20)	**750**	75 ± 10 (0.64)	**187.5**	137 ± 15 (1.04)	**75**	359 ± 22 (1.16)	272 ± 37 (0.89)	134 ± 14 (1.09)	164 ± 24 (1.40)
**1000**	10 ± 2 (0.87)	**250**	15 ± 3 (0.97)	**1000**	77 ± 6 (0.65)	**250**	142 ± 8 (1.08)	**100**	345 ± 19 (1.11)	309 ± 26 (1.01)	119 ± 19 (0.96)	179 ± 25 (1.53)
**C +**	635 ± 46 ^a^	**C +**	1079 ± 91 ^d^	**C +**	1226 ± 42 ^b^	**C +**	1970 ± 122 ^d^	**C +**	1982 ± 103 ^c^	1675 ± 85 ^e^	1228 ± 52 ^a^	1952 ± 73 ^d^

**(d):** 

***Copaifera langsdorffii*** ***(oleoresin)***	**Number of revertants (M ± SD)/ plate and MI**							
	**TA98**		**TA100**		**TA97a**		**TA102**	
***μ*** **g/plate**	**-S9**	**+S9**	**-S9**	**+S9**	**-S9**	**+S9**	**-S9**	**+S9**

**C-**	17 ± 4	21 ± 3	117 ± 11	105 ± 9	125 ± 17	132 ± 21	259 ± 40	301 ± 31
**DMSO**	18 ± 2	22 ± 4	126 ± 2	118 ± 12	117 ± 8	150 ± 14	233 ± 25	265 ± 36
**500**	18 ± 3 (1.01)	22 ± 3 (1.02)	96 ± 16 (0.76)	125 ± 6 (1.06)	81 ± 5 (0.69)	126 ± 18 (0.84)	181 ± 17 (0.78)	261 ± 12 (0.99)
**1000**	17 ± 2 (0.95)	23 ± 5 (1.03)	97 ± 13 (0.77)	124 ± 14 (1.06)	84 ± 13 (0.72)	113 ± 15 (0.76)	146 ± 13 (0.63)	215 ± 24 (0.81)
**2000**	16 ± 5 (0.93)	22 ± 5 (1.00)	94 ± 20 (0.75)	129 ± 9 (1.10)	69 ± 8 (0.59)	110 ± 6 (0.74)	144 ± 14 (0.62)	213 ± 26 (0.80)
**3000**	15 ± 1 (0.83)	22 ± 2 (1.02)	66 ± 12 (0.52)	106 ± 15 (0.90)	73 ± 3 (0.62)	82 ± 4 (0.55)	131 ± 8 (0.56)	128 ± 11 (0.48)
**4000**	13 ± 2 (0.74)	23 ± 8 (1.06)	61 ± 11 (0.48)	112 ± 11 (0.95)	54 ± 6 (0.46)	83 ± 2 (0.55)	133 ± 11 (0.57)	138 ± 15 (0.52)
**C+**	651 ± 42 ^a^	1115 ± 56 ^d^	1123 ± 85 ^b^	1256 ± 93 ^d^	1024 ± 73 ^a^	1672 ± 43 ^d^	1015 ± 95 ^c^	1825 ± 81 ^e^

**(e):** 

**Kaurenoic acid**	**Number of revertants (M ± SD)/ plate and MI**							
	**TA98**		**TA100**		**TA102**		**TA97a**	
***μ*** **g/plate**	**- S9**	**+ S9**	**- S9**	**+ S9**	**- S9**	**+ S9**	**- S9**	**+ S9**

**C-**	20 ± 3	15 ± 1	125 ± 14	114 ± 10	310 ± 35	275 ± 23	128 ± 12	134 ± 17
**DMSO**	13 ± 4	15 ± 2	108 ± 9	100 ± 6	310 ± 12	303 ± 14	123 ± 13	117 ± 25
**25**	15 ± 3 (1.15)	17 ± 2 (1.10)	91 ± 8 (0.85)	92 ± 1 (0.91)	246 ± 11 (0.79)	332 ± 11 (1.10)	124 ± 22 (1.00)	130 ± 11 (1.10)
**50**	15 ± 4 (1.12)	16 ± 4 (1.07)	94 ± 2 (0.87)	98 ± 16 (0.97)	291 ± 16 (0.94)	307 ± 21 (1.01)	113 ± 20 (0.92)	133 ± 6 (1.13)
**100**	15 ± 5 (1.12)	17 ± 4 (1.10)	94 ± 8 (0.87)	99 ± 13 (0.99)	285 ± 13 (0.92)	336 ± 20 (1.11)	110 ± 18 (0.89)	96 ± 8 (0.81)
**150**	17 ± 1 (1.31)	15 ± 4 (1.00)	98 ± 7 (0.91)	102 ± 4 (1.02)	301 ± 11 (0.97)	280 ± 31 (0.92)	111 ± 15 (0.90)	81 ± 2 (0.69)
**200**	17 ± 4 (1.27)	13 ± 3 (0.87)	94 ± 6 (0.87)	110 ± 2 (1.09)	299 ± 24 (0.97)	277 ± 20 (0.91)	111 ± 12 (0.90)	68 ± 4 (0.58)
**C +**	435 ± 26 ^a^	809 ± 31 ^d^	1539 ± 82 ^b^	1021 ± 75 ^d^	1982 ± 103 ^c^	2359 ± 201 ^e^	1228 ± 52 ^a^	1952 ± 73 ^d^

^*∗*^ *p* < 0.05 (ANOVA); ^*∗∗*^*p* < 0.01 (ANOVA); M ± SD = mean and standard deviation; Negative Control: rate of spontaneous reversion; Solvent Control: dimethyl sulfoxide (DMSO, 100 *μ*L/plate); Positive Control (C+); a 4-nitro-o-phenylenediamine (10.0 *μ*g/plate, TA98 and TA97a); b sodium azide (1.25 *μ*g/plate, TA100); c mitomycin (0.5 *μ*g/plate, TA102), in the absence of S9; and d 2-anthramine (1.25 *μ*g/plate, TA98, TA100, and TA97a); e 2-aminofluorene (10.0 *μ*g/plate, TA102), in the presence of S9. Values in brackets (MI) ≥2 indicate mutagenicity.

## Data Availability

The data used to support the findings of this study are included within the article.
